# Cancer-Associated Fibroblast Functions as a Road-Block in Cancer Therapy

**DOI:** 10.3390/cancers13205246

**Published:** 2021-10-19

**Authors:** Pradip De, Jennifer Aske, Nandini Dey

**Affiliations:** Translational Oncology Laboratory, Avera Cancer Institute, Sioux Falls, SD 57105, USA; Pradip.De@avera.org (P.D.); Jennifer.Aske@avera.org (J.A.)

**Keywords:** cancer-associated fibroblasts, tumor microenvironment, cancer therapy, immune cells, stress, extracellular matrix

## Abstract

**Simple Summary:**

An overwhelming percentage of deaths in solid tumors are caused by treatment failure due to the disease’s unresponsiveness when tumor cells tolerate treatment. Aggressive cancer contains tumors cells that are surrounded by many other non-tumor cells, including fibroblasts cells. These fibroblasts near tumor cells are converted by the tumor cells into specialized fibroblasts called cancer-associated fibroblasts that favor the growth of tumors. This review examines how cancer-associated fibroblasts interact with tumor cells, immune cells, and endothelial cells in aiding and abetting the development of resistance to different types of cancer therapy. As cancer-associated fibroblasts’ function blocks the road to recovery, we need to neutralize their function for the clinical management of the disease to be successful. The knowledge about the role of cancer-associated fibroblasts in resisting therapy is fundamental to design an appropriate remedy to counteract drug resistance and improve the outcome of the disease.

**Abstract:**

The journey of a normal resident fibroblast belonging to the tumor microenvironment (TME) from being a tumor pacifier to a tumor patron is fascinating. We introduce cancer-associated fibroblast (CAF) as a crucial component of the TME. Activated-CAF partners with tumor cells and all components of TME in an established solid tumor. We briefly overview the origin, activation, markers, and overall functions of CAF with a particular reference to how different functions of CAF in an established tumor are functionally connected to the development of resistance to cancer therapy in solid tumors. We interrogate the role of CAF in mediating resistance to different modes of therapies. Functional diversity of CAF in orchestrating treatment resistance in solid tumors portrays CAF as a common orchestrator of treatment resistance; a roadblock in cancer therapy

## 1. Background of an Established Tumor

Once a tumor has been established, its metastatic progression in spite of any treatment is the single most critical determinant of the dismal outcome of the disease. The growth and metastatic progression of cancer are functions of the genomic characteristics of tumor cells. Although genetic alteration(s) defines the fate of the tumor and prognosis in patients, cancer does not grow in a void inside the body. The extra-tumoral, non-transformed, immediate tumor-adjacent component of the host, which connects neoplastic cells to the host, is called the tumor microenvironment (TME). A tumor is a product of transformed self-cells within the host body. An established tumor behaves as a self-declared autonomous entity contradictory to the host’s health system. At a particular stage of growth of a tumor, tumor cells with a characteristic genomic background start influencing their microenvironment to favor their proliferation and metastasis-associated phenotypes via establishing TME-tumor cross talk. These critical events thus determine the fate of the tumor and the clinical outcome of the patient. The specialized niche within TME provides metabolic support, immune surveillance, angiocrine, and inflammatory milieu to the tumor cells as a host. TME is, therefore, crucial to determine a tumor’s clinical fate, its growth, its progression through the course of treatment, and finally, its response to a drug. Thus TME is a solid tumor’s deterministic and dynamic context [1,2]. In the following section, the importance of TME will be interrogated in the context of its relationship with tumor cells. We seek to understand the role of TME and its components because it determines the characteristics of the tumor progression and the response of the tumor to treatment and outcome.

## 2. Relationship of Cancer-Associated Fibroblasts and Tumor Cells in Tumor’s Microenvironment: A Turning Table

**Figure 1:**
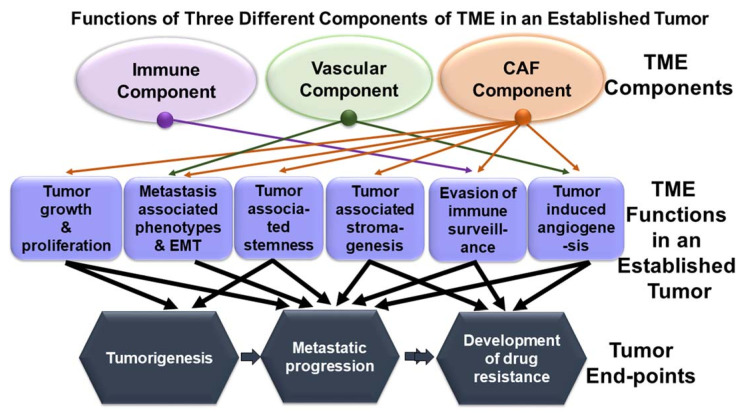
CAF component of TME plays a critical role in promoting tumorigenesis and determines tumor fate in solid tumors. The relationship between TME components (CAF compartment, immune compartment, and vascular compartment; as represented in oval shapes), TME functions (as represented in a square), and tumor end-points (as illustrated in hexagonal shapes) are diagrammatically presented. The diagram is presented in three layers. The top layer presents three TME components, the middle layer presents various functions of the CAF compartment, immune compartment, and vascular compartment in an established tumor, and the bottom layer presents tumor end-points. In an established tumor, TME influences (1) tumor growth and proliferation, (2) metastasis-associated phenotypes and EMT, (3) tumor-associated stemness, (4) tumor-associated stromagenesis, (5) evasion of immune surveillance, and (6) tumor-induced angiogenesis. The immune component is involved in the evasion of immune surveillance of the tumor cells (violet arrow), while the vascular component is involved in tumor-induced angiogenesis and thus influences metastasis-associated phenotypes (Green Arrow). Unlike the other two components of TME, it is noteworthy that the CAF component of TME plays a critical role in promoting every function of TME (orange arrow). Black arrows connect various functions of TME with tumor end-points. The diagram attempts to visualize how CAF influences tumor end-points, which in turn determines the clinical outcome of a disease. Tumorigenesis may lead to metastatic progression (single arrow) and/or may exhibit resistance to drug (double arrow), leading to a dismal clinical outcome.

The TME of an established tumor is composed of three broad components, immune component, microvascular endothelial/lymphatic component, and fibroblast/mesenchyme component that together comprise the stromal microenvironment [3,4,5]. The tumor-TME relationship is unique. At a given time, all three components of TME interact with the tumor both directly and via inter-component crosstalk. A tumor cell’s interaction with the host’s extra-tumoral stroma plays a deterministic role in the process of (1) metabolic and stromal support for tumorigenesis, (2) stemness and metastatic progression, and (3) response to various treatment modalities. Together, all three components of TME facilitate proliferation and the ensuing metastasis-associated phenotypes, perturbs immune surveillance and supports tumor cells in their effort to evade immune recognition, and actively participates in developing drug-induced resistance in cancer cells which hinders disease management. Such an array of effects in real-time is known to be achieved via multi-directional and multimodal TME-tumor crosstalk, orchestrating various signals for different tumor phenotypes (Figure 1). Figure 1 shows the multilayered relationship between TME components and the clinical outcome of disease in an established tumor. Figure 1 is a schematic representation of functions (square shapes) of CAF as a component of TME (oval shapes) in an established tumor. The diagram connects the functions of different components of TME to their various functions which are involved in tumor end-points (hexagonal shapes), including tumorigenesis, metastatic progression, and development of drug resistance. Tumorigenesis may lead to metastatic progression (single arrow), and in the worst-case, develop resistance to drug (double arrow), leading to a dismal clinical outcome.

## 3. CAF in an Established Tumor

A tumor contains cancer cells and stromal cells. The TME is formed by soluble and insoluble factors provided by both cancer and stromal cells. Within an established tumor, the TME represents an altered part of the original normal parenchymal tissue of the host; the change is mainly contributed by the tumor cells in their favor. This stromal transformation of TME, the physical, architectural, cyto-immune, and secretory, called “stromagenesis” is predominantly initiated and maintained by CAFs. TME reflects the functions of CAFs [6]. Considering the importance of CAF in determining the outcome of clinical management of solid tumors, interests have been renewed in targeting CAF [7]. The presence of CAF is identified in almost all solid tumors, including rare cancers such as cholangiocarcinomas [8]. Studies have indicated that the CAF component of TME is the most critical in influencing (directly or indirectly) almost all the functions of TME in real-time [9,10,11]. Crosstalk of CAFs with tumor as well as other TME components co-evolve with tumor progression and treatment, thus connecting CAF to the ultimate clinical outcome of the disease.

The role of CAF in the biology of evolution and clinical outcome of a solid tumor is both supportive and deterministic. Tumor-CAF interaction’s most fascinating characteristic is that it is bi-directional, dynamic, and adaptive [12,13,14]. Tumors evolve with time and treatment. So evolves the interaction of CAF with tumor and with every component of TME, turning the table in favor of tumor progression. The evolving temporal-spatial interaction of tumor cells carrying a set of genomic characteristics with its CAF determines its progression and response/resistance to the drug. Based on the number of functions of CAF, here we interrogate the role of CAF in mediating the development of resistance in solid tumor cells to different therapies. We connect various actions of CAF responsible for the development of different types of resistance in solid tumors and finally discuss the futuristic implication of this understanding from the therapeutic standpoint.

## 4. Definition of CAF

CAFs can be defined as the mesenchymal fibroblastic component of TME in established solid tumors [3]. CAFs are the frequent most non-neoplastic cellular component of TME. The origin of CAF can explain the dichotomy of the definition of CAF. CAFs are a heterogeneous and dynamically plastic mesenchymal cell population-specific to a particular organ type of cancer [15,16]. The study of the origin of CAF in a particular organ type is challenging, and so are their consensus markers and their modes of activation. CAFs can originate from normal tissue-resident fibroblasts (nonactivated and quiescent tissue-resident fibroblasts). CAFs can also originate from a number of non-fibroblastic mesenchymal cells. There are many types of cells that can give rise to CAFs, as reported in several in vitro and in vivo studies [3,17,18,19,20]. CAFs are formed (inducted) from their normal tissue-resident fibroblasts or non-fibroblastic mesenchymal elements by tumor cells via multiple mechanisms, including (1) contact signals between cancer cells and fibroblasts via Notch and Eph–ephrins, (2) RTK ligands like PDGF and FGF, (3) physical and chemical changes in the extracellular matrix, (4) IL-1, IL-6 and TNF mediated inflammatory signals, (4) chemotherapy and radiotherapy mediated DNA damage, (5) heat shock factor1, ROS, and metabolic stress, (6) TGFβ family ligands and the lipid mediator lysophosphatidic acid, (7) JAK-STAT signaling, (8) alterations in epigenetic histone acetylation, fibroblast stretching, and (9) SRF-dependent and YAP1-dependent transcriptional programs. The detailed CAF activation mechanism is reviewed elsewhere [21,22,23]. The definition of CAF is self-explanatory for the heterogeneity of CAFs and their markers. In the following sections, the heterogeneity of CAFs, and their markers are presented.

## 5. Heterogeneity of CAF

The heterogeneity of CAF is the product of its origin and factor(s) that reprogram its conversion in a specific organ-type cancer. CAF can originate from: (1) resident fibroblast, (2) fibrocytes, (3) myeloid cells, (4) pericytes, (5) endothelial cells, (6) adipocytes, (7) epithelial cells, (8) mesenchymal stem cells, (9) stellate cells and (10) mesothelial cells. If the origin of CAF is not heterogeneous enough, their reprogramming factors, including: (1) stromal secretomes, (2) epigenetic, (3) miRNAs, (4) existing CAF, (5) tumor secretomes, (6) metabolic reprogramming, (7) oxidative stress, (8) hypoxia, (9) shear stress within ECM, and (10) indirect effect of radio/drug therapy add to the heterogeneity. The diversity of the origin and modes of origin of CAF presented above explains the possibility of its spatial heterogeneity and a simultaneous presence of multiple CAF subpopulations across solid tumors [14,16,20,24,25,26]. Also, the diversity of their origin can be associated with their population subsets within a tumor, their markers, their biology, and hence their specific functions [13,27]. One example of CAF sub-population is found in pancreatic adenocarcinomas (PDAC). PDAC presents inflammatory cancer-associated fibroblasts (iCAFs), myofibroblastic CAFs (myCAFs), and antigen-presenting CAFs (apCAFs). The above-mentioned complexity in cellular and functional heterogeneity of CAF forms the background of the phenomenon of ‘stromagenesis’ and “stromal homeostasis” in solid tumors [6,20,28]. Stromagenesis refers to multiple states comprising of a complex network of bi-directional stromal fibroblastic signaling pathways and tumor-induced pro-neoplastic modification of stromal extracellular matrix [28]. Stromagenesis is often found to be coupled to tumorigenesis [28], which generates pro-tumorigenic CAF from tumor-suppressive initial fibroblastic reactions within a host tissue [29,30]. Whether tumor heterogeneity can be indirectly connected and explained by the temporal-spatial heterogeneity of a simultaneous presence of different sub-populations of the CAFs within a single tumor at a particular time/stage of the disease remains to be conclusively proved. If true, this fact can represent an intra-tumoral CAF-tumor cells ecosystem. And explain why and how CAFs co-evolve with tumor cells during the metastatic progression of the disease and the development of drug resistance during the clinical intervention of the disease, the single most critical determinants of the disease outcome.

## 6. Markers of CAF

It is not surprising that the markers for CAF are related to their cell of origin and their diverse modes of origin in a particular organ-type tissue. The current hypotheses include the conversion of adipocytes, pericytes, endothelial cells, bone marrow-derived mesenchymal stem cells, and mesenchymally transformed tumor epithelial cells into CAFs. The conclusion is further challenged by the lack of knowledge of whether: (1) individual CAF populations at a particular time are preserved across tissues and species of laboratory animals, (2) a particular CAF subtype is influenced and altered in real-time by spatial location within a heterogenous and evolving tumor, and (3) a particular divergent CAF phenotype is generated locally by a particular mechanism of activation. The CAF markers are (1) surface markers (fibroblast activation protein, podoplanin, platelet-derived growth factor receptor-alpha, platelet-derived growth factor receptor-beta), (2) intracellular markers (alpha-SMA, vimentin, fibroblast specific protein 1), and (3) extracellular markers (lumican, decorin, collagen1A1, collagen1A2) [13]. Not unexpected is the heterogeneity of CAF subtypes across various organ-type malignancies including, head and neck cancer, lung cancer, PDAC, melanoma, breast cancer, and colon cancers. It is worth mentioning that, unlike other solid tumors, there is a conspicuous absence of data regarding CAF heterogeneity and markers for primary gynecological cancers. The markers of CAFs, in general, are reviewed elsewhere [3,21,31].

Considering the heterogeneity and plasticity, CAFs can be functionally classified into two broad states based on their phenotypes, markers, and signaling as (1) myofibroblast CAF and (2) inflammatory CAF in PDAC as mentioned before [32,33]. Myofibroblast CAFs are of the myofibroblast origin, expressing high TGFbeta-driven SMA-α phenotype and contractile property. In contrast, inflammatory-CAF exhibits a high IL-6 secretome phenotype which is exclusively negative for alpha-SMA [34]. The classification of CAF is evolving. Recently, two new subtypes have been proposed including, (1) antigen-presenting CAFs (apCAF) expressing major histocompatibility complex (MHC) class II genes associated with CD4+ T-cells deactivation, and (2) meflin, a membrane-anchored protein, -expressing CAFs (meflin-CAF), that reduces tumor progression [35]. The existence of the default heterogeneity of markers of CAF can be attributed to (1) overlap of markers, (2) organ-type specific markers of CAF, and (3) overlap of marker-receptors of CAF such as PDGFRα/β, TGFβRI/II, EGFR, FGFR, BMPRI/II and their specific signals with the same receptor-mediated signals within cancer cells. Such a problem can be approached using: (1) multiple markers and (2) isolating and primary culturing of CAF through multiple passages. The default heterogeneity of the origins and markers of CAF in different organ-type cancers argues that the biology and mode of functions of CAF are specific to a particular organ-type cancer.

## 7. Biology and Functions of CAF in Solid Tumors

The biology and function of CAFs are as diverse as their cells of origin, modes of origin, and sub-populations/specialization [12,13,14]. The principal functions of CAFs include: (1) supporting proliferation and growth of tumor cells, (2) initiating metastasis-associated phenotypes, (3) orchestrating immune evasion for tumor cells, (4) initiating peri-tumoral matrix remodeling/stromagenesis, (5) angiogenesis, (6) metabolic reprograming of TME, (7) facilitating stemness and the development of drug resistance, and (8) progression of metastatic disease. The types of functions of CAF are multimodal and change in real-time depending on the evolution of the tumor. The detailed nature of the function of CAFs, as maintained via its crosstalk with tumor cells in the context of tumor growth/progression, immune evasion, is reviewed by others [20,21,36,37,38].

As the functional classification is still in its early days, the sub-types are more organ type-specific like in PDAC, prostate cancers, GI cancers (gastric cancer, colorectal cancer, rectal cancer, esophageal cancer), gynecological malignancies and breast cancers [39,40,41,42,43]. In breast cancers, divergent CAF phenotypes have been reported in FAP-positive CAFs. FAP-high CAFs are correlated with Treg cell-mediated immunosuppression and thus a poor outcome [44]. In a model of human pancreatic cancers, targeting CXCL12 from FAP-expressing CAF has been shown to synergize with anti- PD-L1 immunotherapy [45]. CAFs have also been reported to promote cancer formation and are involved in chemoresistance by sustaining cancer stemness in breast cancers [46]. In colon cancers, the Wnt-pathway-induced functional diversity of colorectal CAF has been shown to represent a non-cell-autonomous mechanism for cancer progression [33]. CAFs have multi-pronged action on ‘stromagenesis’ and they crosstalk with tumor cells [47]. CAFs regulate both the biochemical and physical structure of the tumor microenvironment as well as its interaction with cancer cells in real-time. The event of ‘stromagenesis’ is initiated and maintained by CAFs via: (1) secreting extracellular matrix components and extracellular vesicles [48], (2) metabolic reprogramming of tumor stroma via metabolic coupling between CAFs and cancer cells [35,49,50,51], and (3) phenotypic plasticity to co-evolve with the progression of the disease and response to therapy [21,52]. Metabolism shapes the TME, and metabolic regulation of tumor cells by the TME creates intratumoral metabolic heterogeneity/reprogramming, which in turn regulates tumor immunology by tumor-secreted metabolites [53,54]. Metabolic remodeling is the way for both tumor cells and CAF to respond and adapt to changes in the TME. Because metabolic adaptation to TME is a determining event in an established tumor by virtue of which tumor cells are able to grow locally, evade the host’s immune defense, invade and metastasize [55], metabolic reprogramming has been one of the emerging concepts associated with the development of resistance to the therapy. As the most versatile and overarching contributor of TME, CAF controls and adapts to a metabolic landscape of TME and its functional consequence on the tumor cells [35,56].

The most intriguing and critical aspect of CAF function is the crosstalk of CAF with tumor cells. It is essential to understand the crosstalk as it provides insight into the future of stroma-based targeting to manage cancers with a dominant stroma-CAF component. Secretion of numerous cytokines by both epithelial tumor cells and CAFs forms the language of this complex paracrine intratumoral dialogue, which controls several phenotypes of a tumor cell, including proliferation, angiogenesis, metabolic reprogramming, immune evasion, immune suppression, metastasis, and chemoresistance [3,57,58]. An excellent example of the crosstalk is presented in the case of PDAC, where tumorigenesis is highly dependent on the establishment of a fibrotic-stroma that provides aberrant interactions of cancer cells with stromal compartments [59]. The extent and depth of integration of CAF with surrounding tumor cells can be understood from the study on the role of p53 mutation on the function of CAF. CAF hierarchy driven by the p53-status of cancer cells to create a pro-metastatic and chemoresistant environment has been identified in PDAC [60]. Interrogating how cancer cells harboring distinct alterations in p53 manipulate CAFs, Vennin et al. identified a p53-driven hierarchy, where cancer cells with a gain-of-function (GOF) mutant *TP53* have been shown to educate a dominant population of CAFs that established a pro-metastatic environment for GOF and null p53 cancer cells alike. CAFs educated by null p53 cancer cells were shown to be reprogrammed by either GOF mutant p53 cells or their CAFs leading to the acquisition of more invasive and metastatic features. These dominant CAFs, in return, delay cancer cell response to gemcitabine/abraxane (irrelevant of the cancer cell p53 status) by creating a protective environment and via direct interactions with the cancer cells [60]. Interestingly, a distinct way of immune suppression by CAFs involving cell-to-cell contacts has been identified in PDAC. Gorchs et al. reported that CAFs induced expression of immune checkpoints on CD4+ and CD8+ T-cells, which diminished immune function. On the other hand, CAFs promoted the expression of TIM-3, PD-1, CTLA-4, and LAG-3 in proliferating T-cells. The residing T-cells within the desmoplastic stromal compartment expressed PD-1, indicating a role for CAFs on co-inhibitory marker expression. In line with the above results, proliferating T-cells expressing immune checkpoints produced less IFN-γ, TNF-α, and CD107a following restimulation in the presence of CAFs [61].

## 8. Connecting Biology and Function of CAF to the Development of Resistance to Different Therapies in Solid Tumors

CAF is an individual entity for cancer progression. In aiding and abetting the progression of a tumor, CAFs play a critical role in: (1) the development of resistance to therapies and (2) overcoming physiological stress of tumor cells at the crossroads of metastasis, stemness, and epithelial-mesenchymal transition (EMT) [62]. CAFs are considered as the drivers of disease progression in a number of solid tumors by remodeling the tumouroid stroma [23,62,63,64,65,66,67,68,69,70,71]. In solid tumors, the contribution of CAF has been reported in the development of resistance to: (1) specific therapies and (2) a wide variety of physiological stresses. Specific therapies include radiotherapy, chemotherapy, targeted therapy, and immunotherapy. Physiological stresses include shear-stress of ECM, immune-surveillance, apoptosis/anoikis/ferroptosis, stemness, and metabolic stress. To orchestrate such a wide range of effects, CAFs employ different modes of action specific to the organ types, types of therapies, and physiological stresses. This is accomplished via the diverse biology of CAFs [13]. CAF directly influences the outcome of the clinical management of cancer via certain biological functions reviewed in the above section. One classic example is the pivotal role of the CAF in desmoplastic stroma-dependent solid tumors. The CAF-mediated modulation of integrins and fibrillar collagen interaction within the reactive stroma of ECM explains TME-mediated tumor growth, angiogenesis, metastasis, immune suppression, and resistance to treatments in desmoplastic breast, lung, and pancreatic cancers [72,73].

Modes of cellular and extracellular signaling involved in the development of CAF-mediated resistances in solid tumors are tabulated (Table 1). Table 1 presents the resistant types, signaling mediators, and types of interaction between CAF, tumor cells, and TME in different organ type cancers (represented with their respective ribbon colors). The types of signaling/interaction between CAF, tumor cells, and TME in various organ type cancers include: (1) paracrine, (2) autocrine-paracrine loop, (3) exosomal cargo for miRNAs, (4) extracellular vesicles cargo, (5) cell-to-cell contact, (6) ECM remodeling/neovascularization/vascular mimicry involving CAF secretome, and (7) immune/metabolic reprogramming. Table 1 presents different categories of therapies in which functions of CAFs are involved in the development of resistance, including radio-, chemo-, targeted-, immuno-, therapies. In addition, they play a critical role in overcoming different physiological stresses like shear stress, apoptosis, or anoikis, metabolic stress, and immunological stress for the tumor cells.

**Figure 2:**
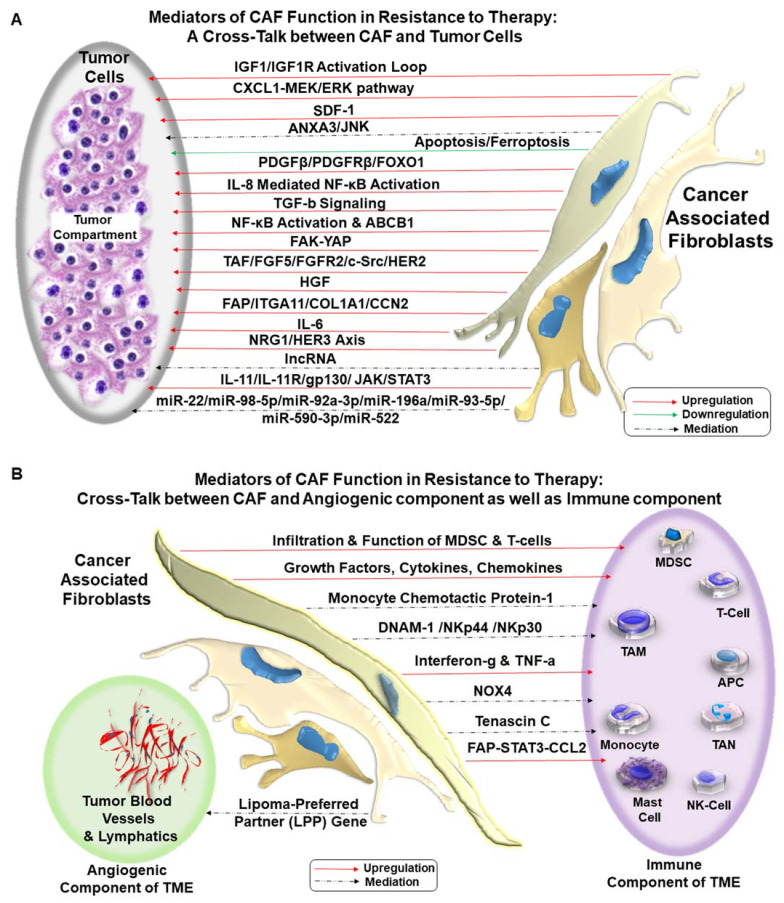
Mediators of CAF functions in the development of resistance to therapy: The development of resistance to various treatments involves dialogues of CAF with (1) tumor cells (CAF-tumor cell crosstalk), (2) tumor-infiltrating immune cells (CAF-immune cell crosstalk), and (3) endothelial cells (CAF-angiogenic cell crosstalk). CAF plays a critical role in the development of resistance to therapy in solid tumors. The CAF-tumor cell crosstalk occurs via several mechanisms involving upregulation (Red Arrow), downregulation (Green Arrow), and complex mediation (Broken Arrow) of signals in different solid tumors. Several cell signaling pathways are upregulated in the CAF-tumor cell crosstalk, including IGF, HGF-cMET, MEK-ERK, JNK, PDGF, NF kappaB, NRG1-HER3, FGFR/c-SRC, and JAK-STAT3 pathways. The crosstalk also involves several miRNAs, including miR-22, miR-98-5p, miR-92a-3p, miR-196a, miR-93-5p, miR-590-3p, miR-522, as well as cytokines like IL-6, IL-11, SDF-1, and TGFbeta. CAF also downregulated apoptosis and ferroptosis of tumor cells (**A**). CAF interacts directly with the angiogenic component and immune component in the development of resistance to therapy in solid tumors. The interaction involves both upregulation (Red Arrow) and complex mediation (Broken Arrow) of signals. CAF influence a number of immune cells, including MDSC, T-cells, tumor-associated macrophages, antigen-presenting cells, monocytes, tumor-associated neutrophils, natural killer cells, and mast cells. The types and mediators of interactions are either specific to an organ-type tumor or common to some solid tumors. The mediators of CAF-immune cells crosstalk are several growth factors, cytokines, chemokines, monocyte chemotactic protein-1, DNAM-1 /NKp44 /NKp30, TNFalpha, interferon-gamma, NOX4, Tenascin C, and STAT3. The interaction of vascular cells and CAF involves the Lipoma-Preferred Partner (LPP) gene (**B**).

It has to be understood that CAFs are non-transformed abettor cells to tumors cells residing in the TME in an established solid tumor. Although the role of epigenetic switch has been suggested in the conversion of fibroblasts into pro-invasive CAF [74], oncogenic mutations in CAF are not proved yet [75]. Thus, as mentioned above, all seven modes of actions or types of interactions between CAF, tumor cells, and TME depend on the spatial orientation of CAF within the tumor mass. Recently, distinct geospatial architectural distributions of CAFs have been reported to be associated with treatment response in renal cell carcinoma (RCC). An ex vivo geospatial analysis of CAF distribution suggested that close proximity clustering of tumor cells and CAFs potentiates tumor cell proliferation resulting in worse OS (overall survival)/poor hazard ratio and resistance to tyrosine-kinase-inhibiting targeted therapies in metastatic clear renal cell carcinoma. Targeted therapy-resistant and immune therapy-resistant patient samples presented a distinct nearest-neighbor (NN) distance from alpha-SMA positive CAFs from proliferating/apoptotic tumor cells [76]. CAFs employ several modes of action in influencing the development of resistance, acting directly or indirectly on: (1) tumor cells, (2) immune cells, and (3) endothelial cells. The development of resistance involves CAF-tumor cell crosstalk via several mechanisms involving multimodal signaling (Figure 2). CAF-tumor crosstalk is presented in Figure 2A. CAF-TME crosstalk is presented in Figure 2B.

## 9. CAFs & Resistance to Radiotherapies

CAFs are inherently resistant to the clastogenic effects of radiotherapy. Radiation fails to cause persistent DNA damage in CAF, and CAFs resist apoptosis following clinically applied doses of radiotherapy [77,78]. In fact, radiation and MAPK inhibition (U0126) have been reported to cause no dose-limiting effect on CAF [79]. The other reason for this differential survival phenomenon is that CAFs exhibit senescence following radiation. Xenograft studies demonstrated that highly plastic CAFs recover from the radiation subsequently, via: (1) autocrine/paracrine secretion of cytokines and growth factors [80], (2) producing a distinct combination of immunoregulatory molecules [78] post senescence, and (3) increasing ability to repair DNA damage and altered indices of oxidative metabolism as demonstrated by increases in protein carbonylation, mitochondrial superoxide anion levels, and modulation of the activity of the antioxidants, manganese superoxide dismutase and catalase. [81]. Recovered CAFs determine the fate of the surviving residual cancer cells, creating a conducive stroma and providing vital support to the tumor compartment [82], leading to radioresistance of tumors. The development of CAF-assisted radioresistance is primarily reported in colorectal, esophageal, and nasopharyngeal cancers (Table 1). Post radiotherapy interaction between CAF and tumor/TME involves paracrine, autocrine, and miRNA-mediated signals. Autocrine/paracrine signals consist of the activation of IGF1R [83], the IL-8 mediated activation of the NF-κB [84], PDGFbeta/PDGFRbeta/FOXO1 signals [85], and CXCL1 mediated activation of the MEK/ERK pathway [86]. CAF-derived exosomal transport upregulates TGFbeta signaling or the CLCA4-dependent PI3K-AKT [87,88,89]. Collectively, the participation of CAF in the post-radiotherapy resistance involves (a) cytoplasmic or transcriptional activation of growth/stemness signals, (b) reduction of DNA damage mediated apoptosis, and (c) the acceleration of DNA damage repair. It might be argued that the resistance can be counteracted by perturbing the contextual functions of CAF.

## 10. CAFs and Resistance to Chemotherapies

The role of CAF in inducing resistance to chemotherapy is the most commonly reported and studied event among all resistances in solid tumors. We have interrogated 16 reports in several organ types of cancers, including colorectal, breast, ovarian, gastric, head-neck, pancreas, lung, and bladder (Table 1). It is known that the development of chemotherapy-induced resistance is dependent on the specific drug used for the therapy, its interaction with the tumor cells, and the adaptive response of the tumor cells in the face of the drug. In line with the fact, most of the mediators of CAF function involved in the development of the resistance has been reported to be primarily via paracrine [90,91,92,93,94,95,96,97] or exosomal delivery of various miRNA [98,99,100,101,102].The paracrine mode of action involved secretion of CAF-derived growth factors, cytokines, chemokines like IL-6, IL-8, IL-11, SDF-1, as presented in Table 1. The secretomic initiation of resistance led to the activation of a number of cell signals in tumor cells in different solid tumors. CAF has been reported to have crosstalk with endothelial cells via a paracrine loop in ovarian cancers involving upregulated the lipoma-preferred partner gene in microvascular endothelial cells [96]. In comparison, cisplatin resistance was induced by plasminogen activator-1 produced by CAFs in lung adenocarcinomas [103]. In contrast to the paracrine mode, which involves several different signals, the exosomal cargo mainly delivered miRNAs. These exosomal miRNAs target different signaling proteins in tumor cells leading to the development of resistance. The type of miRNA delivered to tumor cells depends on the organ type cancers, such as miR-196a in head-neck cancers, miR-522 in gastric cancers, miR-92a-3p in colorectal cancers, and miR-98-5p in ovarian cancers. And hence they initiated different cellular signaling in individual tumor cells leading to resistance to various chemotherapies. Gastric cancers represent the most commonly reported cancers that exhibit CAF-induced resistance. In addition to exosomal cargo, gastric cancers are reported to use extracellular vesicles cargo. Exosomes are carriers of cellular proteins, mRNA, miRNA, lipids like microvesicles. Exosomes are the smallest vesicles (30–100 nm) released by fusing multivesicular bodies with the plasma membrane containing intraluminal vesicles, while microvesicles are outward blebbing (100–1,000 nm) of the plasma membrane [104]. Extracellular vesicles cargo has been reported to either deliver miR-522 involving activation of USP7/hnRNPA1 axis [99] or via annexin A6 activating FAK-YAP signaling [105].

## 11. CAFs and Resistance to Targeted Therapies

Evidence suggests that the CAF-induced development of resistance to the targeted therapies is limited in terms of organ type and treatment modalities. The reported organ types include breast [106,107], lung [108,109], prostate [110], hepatocellular carcinoma [111], and melanoma [112] (Table 1). Unlike chemotherapy resistance, the modes of action of CAF in developing resistance to targeted therapy involve predominantly paracrine interaction via secretomes. However, an exosomal cargo-based delivery of miRNAs, miR-22 in breast cancer, has been reported [106]. A combination of paracrine mode with a cell-to-cell contact is reported in hepatocellular carcinomas [111].

It is to be understood that CAF performs an array of functions via communicating with both tumor cells and cellular components of TME [113] in bringing the resistance to the drug. CAF’s specific set of functions for the purpose is determined by the organ type and drug against which the resistance is achieved. Such an argument is strengthened by the report from the group of Prof. Erik Sahai on the action of CAFs in conferring resistance to targeted therapies; resistance acquired by BRAF-mutated melanoma cells following PLX4720 [114]. Sahai et al. identified a drug-tolerant TME in PLX4720 (a potent and selective inhibitor of B-RAFV600E; a progenitor of PLX4032, vemurafenib) treated BRAF-mutated melanoma cells using intravital imaging. In response to PLX4720, a drug-tolerant/resistant niche is formed in areas of high stromal density due to a rapid reactivation of the ERK/MAPK pathway. The crux of this resistance is the PLX4720-mediated activation of melanoma-associated fibroblasts (MAF) which confers tolerance to BRAF inhibition via a switch from BRAF-dependent ERK signaling to BRAF-independent ERK signaling in tumor cells. BRAF inhibition following PLX4720 “paradoxically” activated MAFs to cause: (a) ERK activation (by Western blot and fluorescence resonance energy transfer (FRET) signals, (b) increased proliferation of MAFs, (c) enhanced matrix remodeling (d) increased phosphorylation of the critical regulator of actomyosin contractility, MLC2/MYL9, (f) induced dynamic protrusions in MAFs and promoted the formation of dense collagen fibril, (g) upregulated thrombospondin-1, tenascin-C, and PDGFRalpha in MAFs, and (h) changed in ECM composition and stiffness. The activated MAF reprogrammed the matrix (fibronectin-rich matrices with 3-12 kPa elastic modulus) to elevate integrin beta1/FAK/Src signaling in melanoma cells in the localized “haven” of hyper-activated MAF. Thus a BRAF independent, FAK-Src-mediated compensatory activation of ERK in melanoma cells directly counteracted the sensitivity to PLX4720. In contrast, the drug sensitivity remained unresisted in the MAF-poor micro-niches within the tumor mass. Eventually, stroma-mediated BRAF-independent resistant melanoma cells emerged. This exquisite and much-commented study [115,116,117] presented the best mechanistic explanation of how CAF resists a particular targeted drug within the tumor mass of a specific type of cancer by virtue of one particular mode of its action. The study contributed to the generation of mathematical modeling as a theoretical framework for developing TME-mediated drug resistance [21,118].

The CAF in the paraneoplastic stroma, resisting anticancer therapy, may be closely associated with the disease’s progression in real-time [14]. The physical force between tumor cells and CAFs involving actomyosin contractility and remodeling of ECM forms the tracks used for the cooperative and collective invasion or migration of cells. The function of CAF in a progressing disease is reported by Labernadie et al. [119], who demonstrated that E-cadherin/N-cadherin junction is mechanosensitive and CAFs exert pulling forces on cancer cells enabling their collective invasion. Their study provided evidence of E-cadherin/N-cadherin junctions in lung adenocarcinoma and vulval squamous cell carcinoma. CAFs are ideal stromal partners to enable collective cancer cell invasion [120] via an intercellular physical force that is transmitted through a heterophilic adherens junction involving E-cadherin on the cancer cell membrane and N-cadherin on the CAF membrane, which can also exhibit the N-cadherin, afadin (AFDN; adherens junction formation factor), and mechanotransduction mediated CAF repolarization [119]. Such adhesion was found to be mechanically active; when subjected to force, it triggered beta-catenin recruitment and adhesion reinforcement dependent on alpha-catenin/vinculin interaction, showing a mechanically functional heterophilic adhesion between CAFs and cancer cells enabling a cooperative tumor invasion. In studying miR-21 promoted activation of CAFs in the development of gemcitabine resistance of PDAC, Zhang et al. demonstrated that CAFs with high miR-21 expression had elevated MMP-3, MMP-9, PDGF, and CCL-7 expression, which promoted the invasion of PDAC cell lines [121].

Several models for studying CAF functions have been established to elucidate the mechanism of interactions between CAF and cancer cells, use the information for drug screening, and know the CAF-TME interaction. The studies involved testing (1) 3D culture system by making use of biomaterials for drug screening [122], (2) a high-throughput workflow to remodeling of ECM-based micro-tissues [123], (3) 3D breast cancer micro-tissue to know the role of TME on the transport and efficacy of free-doxorubicin [124], and (4) 3D physio-mimetic interpenetrating network-based platform to decode the pro and anti-tumorigenic properties of CAF [125].

## 12. CAFs and Resistance to Drug-Induced Ferroptosis

Very recently, CAFs have been demonstrated to produce resistance to the therapy via blocking ferroptosis. Ferroptosis is a type of regulated necrosis which occurs in cells independent of caspase activity and receptor-interacting protein 1 (RIPK1) kinase activity. During ferroptosis, unlike apoptosis, necroptosis, and pyroptosis, cells die following iron-dependent lipid peroxidation, antagonized by glutathione peroxidase 4 (GPX4) and ferroptosis suppressor protein 1 (FSP1) [126]. As tumor cells mechanistically escape apoptosis/necroptosis, they maintain/ acquire sensitivity to ferroptosis. Understandably, the role of ferroptosis has been implicated in mediating acquired drug resistance, metabolic reprogramming, and immune evasion [127]. Zhang et al. reported a study demonstrating that CAFs secrete exosomal miR-522 to inhibit ferroptosis in gastric cancer cells by targeting ALOX15 and blocking lipid-ROS accumulation [99].

## 13. CAFs and Angiogenic-Resistance

Resistance to anti-angiogenic therapy has a direct relationship to tumor revascularization and relapse. Revascularization constitutes sprouting angiogenesis, vessel co-option, intussusceptive angiogenesis, and vasculogenic mimicry. All of these are contributed by pro-angiogenic cytokines (vascular endothelial growth factor, angiopoietins, matrix metalloproteinases), heterogeneity in tumor-associated endothelial cells, crosstalk with tumor and CAFs, exosomes, extracellular vesicles, matrix stiffness, and contributions from fibroblasts [128]. Thus CAF is a part of an integrated communication network comprising of tumor cells and TME. Recent insights into the role of TME in the development of resistance to anti-angiogenic therapy in solid tumors came from a characteristically vascular tumor, renal cell cancers [129]. The inhibition of vessel formation by anti-angiogenic therapies has been implicated in recent years in addition to immune therapies only to identify resistance-associated with therapy due to a highly dynamic, adaptive, and heterogeneous tumor microenvironment [129]. Marsden et al. demonstrated a relationship between hypoxia, HIF-1alpha, and CAF [130]. HIF-1alpha supports CAF-induced matrix remodeling and cancer cell invasion. Loss of prolyl hydroxylase domain protein (2PHD2) in CAFs phenocopied hypoxia. Tumor microenvironment dynamics in clear cell RCC is directly associated with prognostic and predictive signatures [131]. Ambrosetti et al. demonstrated that CAF functions are a critical determinant of prognosis and resistance to anti-angiogenic therapy in renal cell carcinoma [132]. Their study showed that VEGFR-TKI promotes the development of CAFs, and CAFs, in turn, favor tumor aggressiveness, metastatic dissemination, and resistance to treatment in RCC. VEGFR-TKI resistance was analyzed on co-cultures of RCC cells with CAFs, and the proportion of intra-tumoral CAFs correlated to shorter disease-free and overall survival. Additionally, CAFs increased migration and decreased the VEGFR-TKI-dependent cytotoxic effect of tumor cells. The desmoplastic stroma of pancreatic ductal adenocarcinoma is another key example. CAFs chiefly contribute to lethality. In the CAF populated stromal microenvironment, CAF interacts with cancer cells to drive progression and chemoresistance. Pausch et al. demonstrated that metastasis-associated fibroblasts promote angiogenesis in metastasized pancreatic cancer via the CXCL8 and the CCL2 axes and their implication in prognosis and resistance to anti-angiogenic therapy has been studied [133]. Thus, whether stromal CAF forms a desmoplastic component or has a pivotal role in revascularization, tumors relapse, and resistance to anti-angiogenic therapy ensues. However, the sequence of events in a tumor may vary based on the organ type of the tumor and its treatment history.

## 14. CAFs and Immune-Resistance

Apart from tumor cells, CAF has been reported to interact most dominantly with the immune compartment of the TME (Figure 2B). CAF phenotype and density predict immunological outcomes [134]. The primary motive of crosstalk between CAF and immune cells [135] in cancer is to (1) block immune surveillance [136] and (2) enhance immune evasion of tumor cells [137]. Thus CAF is a crucial determinant of tumor immunity and immunotherapy [138]. Among the immune cells of the immune compartment of TME, CAF has been reported to interact with T-cell, myeloid-derived suppressor cell (MDSC), tumor-associated macrophage (TAM), antigen-presenting cells (APCs), and natural killer (NK) cells.

Successful PD-1-PD-L1-based immunotherapy depends on the availability of healthy and active CD8+ T effector cells within TME close to tumor cells. CAF has been reported to attenuate the immune surveillance within TME via both paracrine and creating a desmoplastic barrier inducing resistance to immunotherapy in several solid tumors. CD8+ T cell clonal proliferation, activation, tumor localization, and cytotoxicity are blocked in the presence of CAFs via suppression of TCR signaling. A recent review by Baker et al. presented an interrogation of the physical and chemical barriers that CAFs raise between effector T cells and their tumor cell targets and its implication on immunotherapies [134]. Work has been presented to show that CAFs recruit and balance CD4+ effector T cell subsets and may drive T helper responses toward a less cytotoxic and Th2-mediated response [134]. CAFs can promote Treg recruitment and differentiation as well as acquire and present antigen to T cells [134]. Studying isolates from breast carcinoma, colorectal carcinoma, and pancreatic ductal adenocarcinoma, Abuwarwar et al. reported that CAFs blocked T-cell proliferation in vitro [139]. In esophageal cancers, CAFs have been reported to regulate immunosuppressive TIL populations and affect intratumoral CD8+ and FoxP3 + T cells via IL6. CD8+ TILs and CAFs were negatively correlated in intratumoral tissues (*P* < 0.001), whereas FoxP3+ TILs were positively correlated (*P* < 0.001) [140]. The role of CAF in the development of resistance to immunotherapy has been presented by Mariathasan et al. [141]. Their study unearthed one of the signaling mechanisms by which CAF attenuates tumor cells’ response to immune checkpoint blocker, atezolizumab, in metastatic urothelial cancers. Examining tumors from a large cohort of patients with metastatic urothelial cancers treated with atezolizumab, Mariathasan et al. identified two major clinical determinants of outcome. Response to treatment was associated with CD8+ T-effector cell phenotype and a high neoantigen/tumor mutation burden.

In contrast, the lack of response was associated with a signature TGF-beta signaling in tumor-associated fibroblasts (CAF). Non-responders showed exclusion of CD8+ T cells from the tumor parenchyma. Instead, CD8+ T cells were found locked in the fibroblast/collagen-rich peritumoral stroma, a characteristic pathological feature encountered in metastatic urothelial cancers. They hypothesized that the stromal barrier’s physical exclusion of T cells limits response to atezolizumab in immune excluded tumors. Using the EMT6 mouse mammary carcinoma model, they sought to determine the role of TGF-beta–activated stroma. Their comprehensive evaluation associated with response and resistance to checkpoint blockade in patients who were treated with an anti-PD-L1 agent (atezolizumab) indicated that TGF-β signaling, which reflected a distinct gene expression signature and involved pSMAD2/3, may counteract anti-tumor immunity by restricting the T-cells in the TME. Accordingly, a co-inhibition of TGF-β and PD-L1 (using their preclinical EMT6 model) reduced TGFβ signaling in stromal cells to facilitate T-cell penetration into the center of tumors, which induced anti-tumor immunity and tumor regression. Tumor regression was associated with the conversion of tumor-phenotype from a “T-cell excluded phenotype” to an “inflamed phenotype,” which can thus synergize with anti–PD-L1 treatment within the tumor-stroma ecosystem. The synergy was linked with the reprogramming of peritumoral stromal fibroblasts (CAF) and increased CD8+ T-effector cells within the tumor, leading to resensitization/anti-tumor immunity to atezolizumab [141].

CAFs also directly participate in the immune function by presenting an antigen(s). Elyada et al. reported the presence of an “antigen-presenting cancer-associated fibroblasts” by cross-species single-cell analysis in PDAC [142]. They corroborated the presence of myofibroblastic CAFs and inflammatory CAFs and defined their unique gene signatures. Elyada et al. described a new population of CAFs that express MHC class II and CD74 but do not express classic costimulatory molecules and termed this cell population “antigen-presenting CAFs”. This CAFs population activates CD4+T cells in an antigen-specific fashion in a model system, confirming their putative immune-modulatory capacity.

CAFs also modulate monocytic functions like regulating monocyte recruitment and differentiation within TME in favor of tumor cells involving the CAF-MDSC axis. CAFs promote immunosuppression by inducing ROS-generating monocytic MDSCs in lung squamous cell carcinomas [136]. Tumor-associated macrophages (TAMs) and CAFs are the main components of infiltrating stromal cells [143] through the ECM, which are reported to collaborate in tumor progression directly. Tumor ECM undergoes continuous remodeling, which is a function of CAF. Notably, a certain immune evasive function of CAFs is known to be mediated via regulation of ECM secretion under the influence of TGF-β signaling [144]. TAMs and CAFs infiltrate into almost all malignant tumors, facilitating cell-to-cell communication. Peripheral blood mononuclear cells (PBMC)-derived macrophages and BM-MSCs (Bone marrow-derived mesenchymal stem cells) are recruited to a tumor site and activated into TAMs and CAFs, respectively, to create a favorable TME for neuroblastoma progression [145]. M2-like macrophages have pro-tumor characteristics that involve the production of angiogenic factors and immunosuppressive molecules. The cell-to-cell interaction/crosstalk between TAM and CAF induced recruitment and activation of each other and their combined activities were associated with the induction of: (1) disease progression, (2) chemoresistance, (3) gene amplification and (4) immunosuppression [143]. Studies revealed that the cellular composition of the principal component cells of stroma differs in each organ-type cancer. The ratio of the density of TAMs to CAFs was higher in tumors of the brain, bone marrow (leukemia), lymph node (lymphoma), kidney, and liver (HCC). In contrast, the ratio of the density of CAFs tends to be higher than TAMs in tumors of the stomach, colon, rectum, lung, liver (CCC), pancreas, and bile duct. While the neuroblastomas, breast, bladder, and prostate expressed similar expression of CAF and TAM [143,145]. However, the clinical relevance of such distribution is awaited, along with the data from gynecological diseases like endometrial and ovarian cancers, which is conspicuously lacking. Although CAFs can interact with different cells of immune components of TME, the most common mode of action of CAF in developing the resistance to immune therapy is paracrine as tabulated in various organ type cancers (Table 1).

## 15. CAF Resisting Apoptosis/Anoikis of Circulating Tumor Cells

At the primary/secondary site of tumors, CAF provides metabolic, immunological, growth, and metastatic provisions for epithelial tumor cells in situ. On the contrary, when tumor cells circulate, as pathologically identified by lymphovascular invasion, LVI, CAF forms clusters with CTC (Circulating Tumor Cells). Cells of epithelial origin suffer apoptosis/anoikis upon losing their attachment. Tumor cells, especially of epithelial origin in circulation, resist apoptosis/anoikis due to the presence of CAF as CAF directly enables the sustenance of CTC in circulation [62,146]. Thus, CAFs actively participate in aiding and abetting CTCs during the metastatic progression of the disease. CAFs form heterotypic clusters with CTCs identified in blood from patients with late-stage diseases in the metastatic setting. Ortiz-Otero et al. demonstrated that “reactive CAFs”, expressing high α-smooth muscle actin and fibroblast activation protein (FAP), disseminate with CTCs in the blood of patients with prostate cancers [147]. These reactive CAFs can induce shear resistance to prostate tumor cells via intercellular contact as well as derived soluble factors to conserve the proliferative capability of tumor cells in the presence of high magnitude fluid shear stress within the blood vessels. The data suggested the use of CAFs as a marker for cancer progression. These actions complement the metabolic reprogramming of CAF, which imparts resistance to metabolic stress and the stress of apoptosis/anoikis of tumor cells.

## 16. Conclusions

We examine the mode of action of CAF in bringing out different types of resistance in several solid tumors in the context of various treatment modalities. The anti-tumor role of a heterogeneous and activated population of CAF in a well-established/progressing tumor is evident from the literature. In contrast, normal tissue fibroblasts are long known to counteract tumorigenesis [148]. The initial debate of “friend-or-foe” based on the disparate set of data [149] on the role of fibroblast can be resolved theoretically. The normal tissue fibroblasts that tend to suppress tumorigenesis are not directly influenced by transformed tumor cells and the ensuing tissue hypoxia due to the rapid growth of the tumor mass. CAFs are activated [150] and, in some instances, reported to be epigenetically driven/reprogrammed by the tumor cells via the pro-inflammatory cytokine, like leukemia inhibitory factor (LIF), into a pro-invasive phenotype [74]. Such an event results in a generation of a heterotypic pool of non-transformed yet converted collection of cells of multiple origins. Thus the homogenous pool of normal tissue fibroblast is far from the heterogeneous pool of activated CAF. CAFs and their long-term impact in an established and progressing tumor cannot be viewed as a function of a normal tissue-resident fibroblast. Once evolved CAFs performs an array of function within TME in concert with tumor cells as presented in the review. The superimposition of Figure 1 on Table 1 reveals the comprehensive preview of different modes of CAF-induced resistances to various therapies in the number of solid tumors via an array of mediators of action as interrogated in the review. A critical evaluation of the functions of CAF, its crosstalk with tumor cells (Figure 2A), and all components of TME (Figure 2B) show an undeniable functional connection between functions of CAF and the development of resistance to different cancer therapies in almost every common solid tumors with a few exceptions of gynecological malignancies. Friend or foe, CAF is neither of the two. Knowing the CAF-tumor crosstalk will empower us to change the treatment strategy from tumor-centric to tumor-TME centric and supplement the targeted/immunotherapy with CAF-directed therapy in favor of a good prognosis. Future studies will let us know the character of organ-type CAF and drug-response specific CAF. Accordingly, the management of the disease can be improved by supplementing the treatment(s) directed towards targeting CAF (“reversal” of CAF phenotype(s) or “normalize” the tumor stroma). In the era of precision medicine, NGS-based genomic information of organ-type CAF may be needed to succeed in tumor-CAF dual-targeted therapy.

## Figures and Tables

**Table 1:**
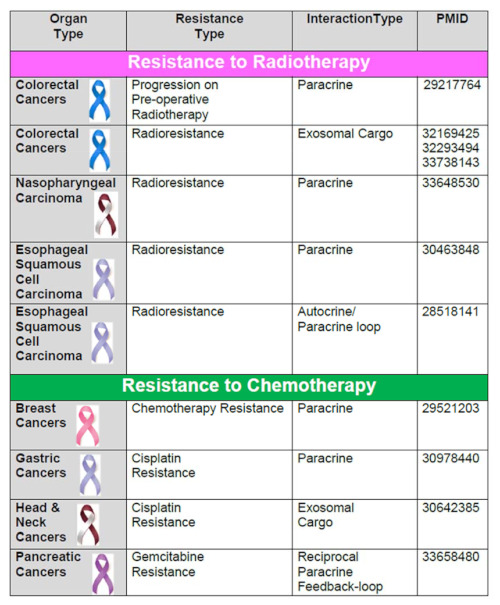

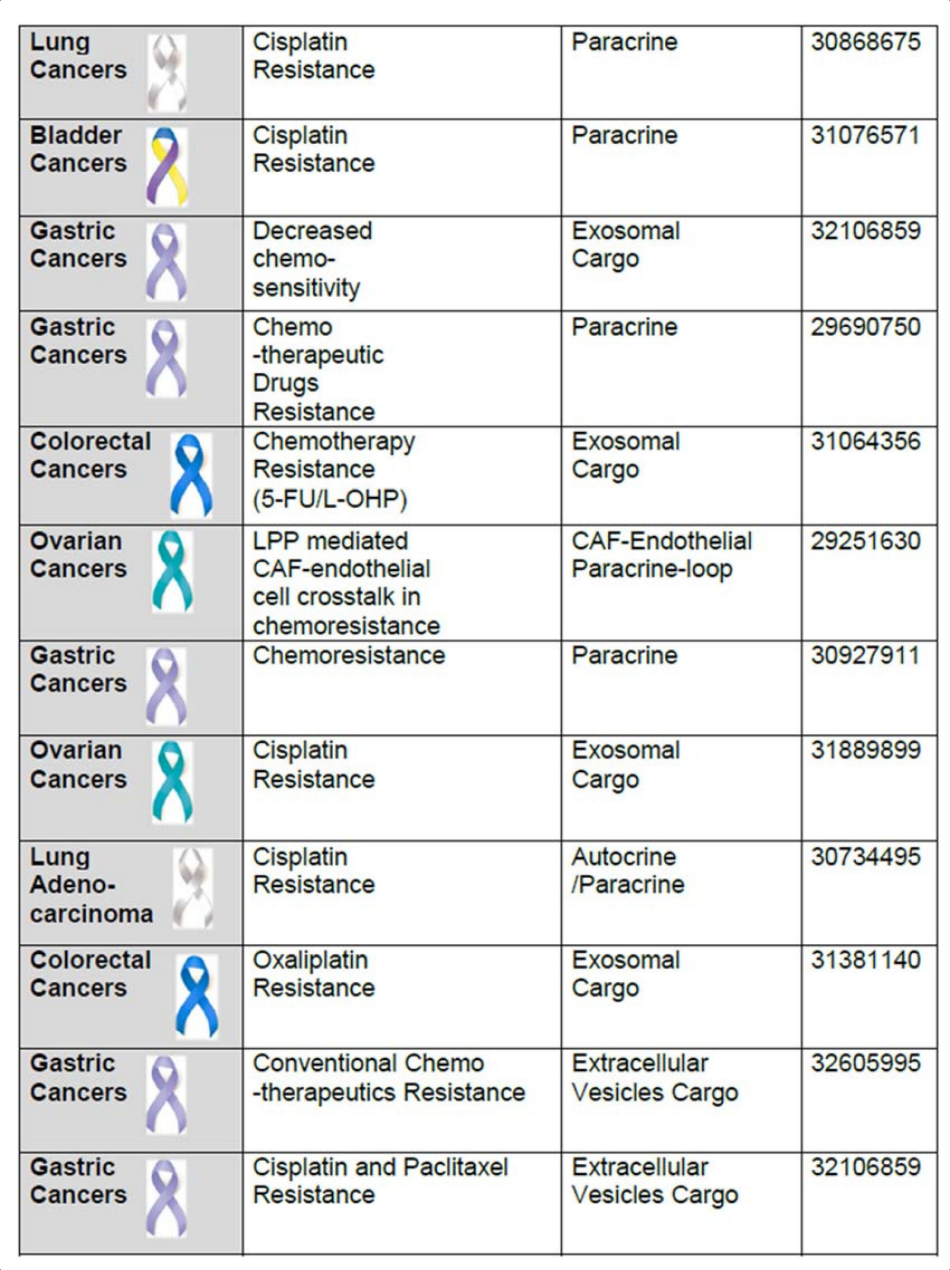

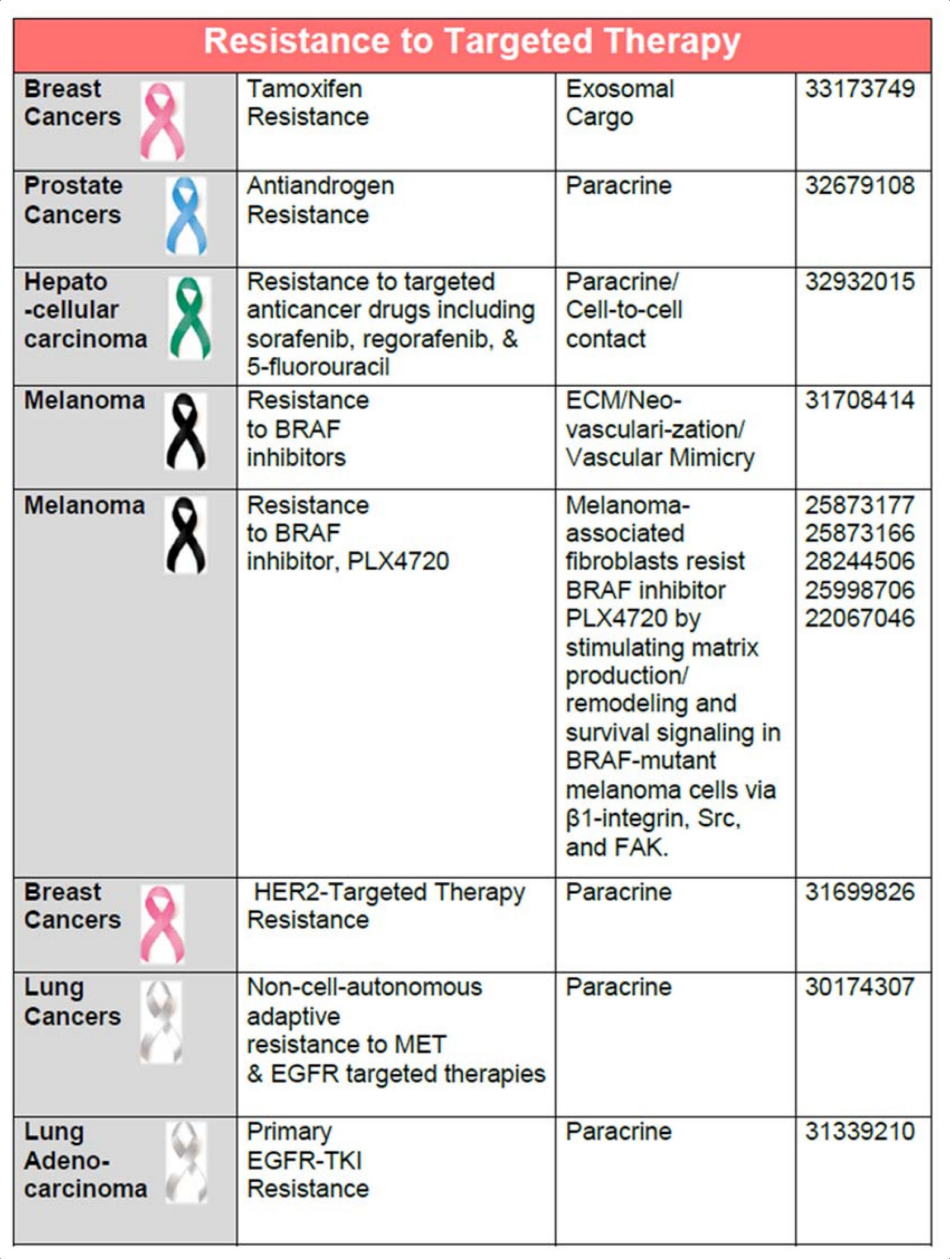

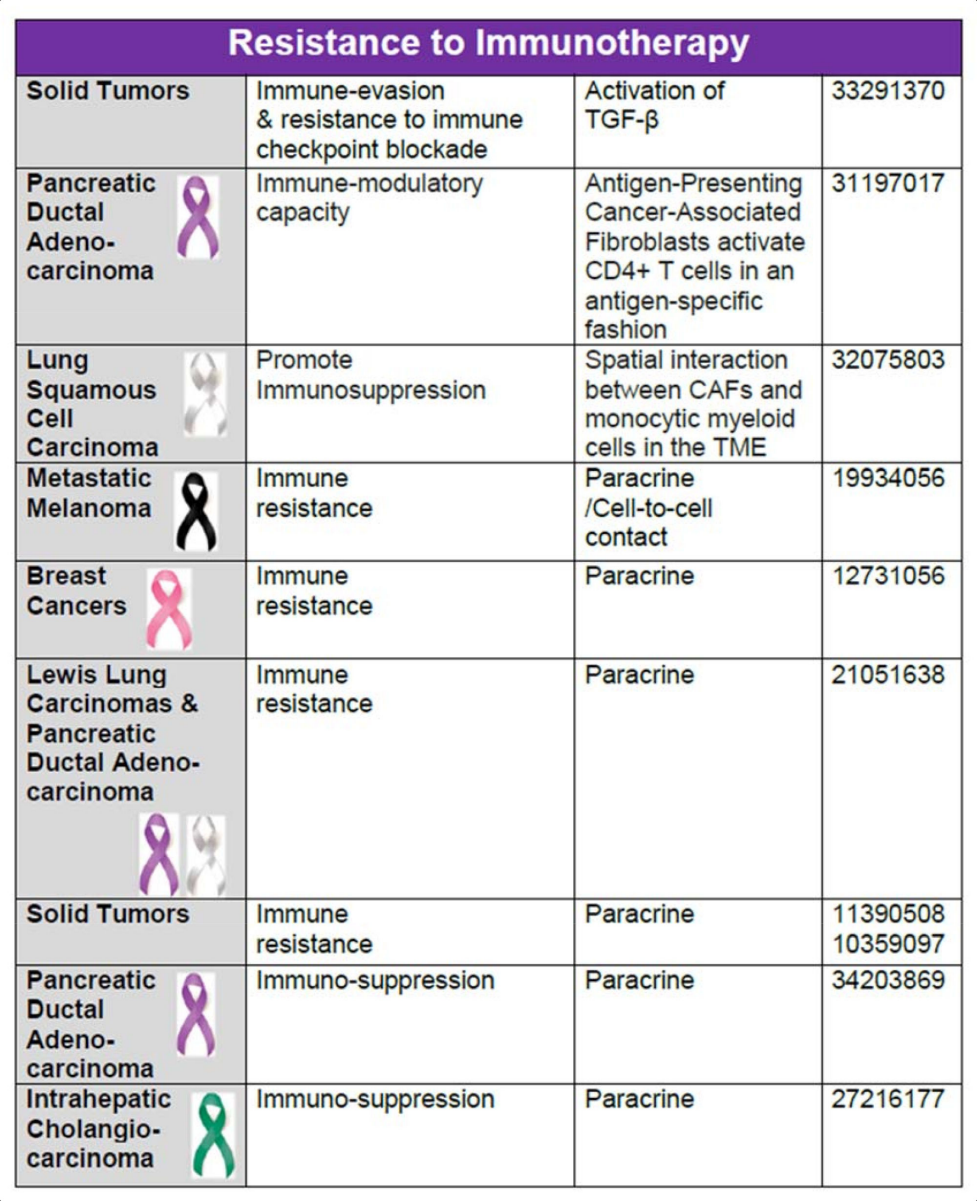

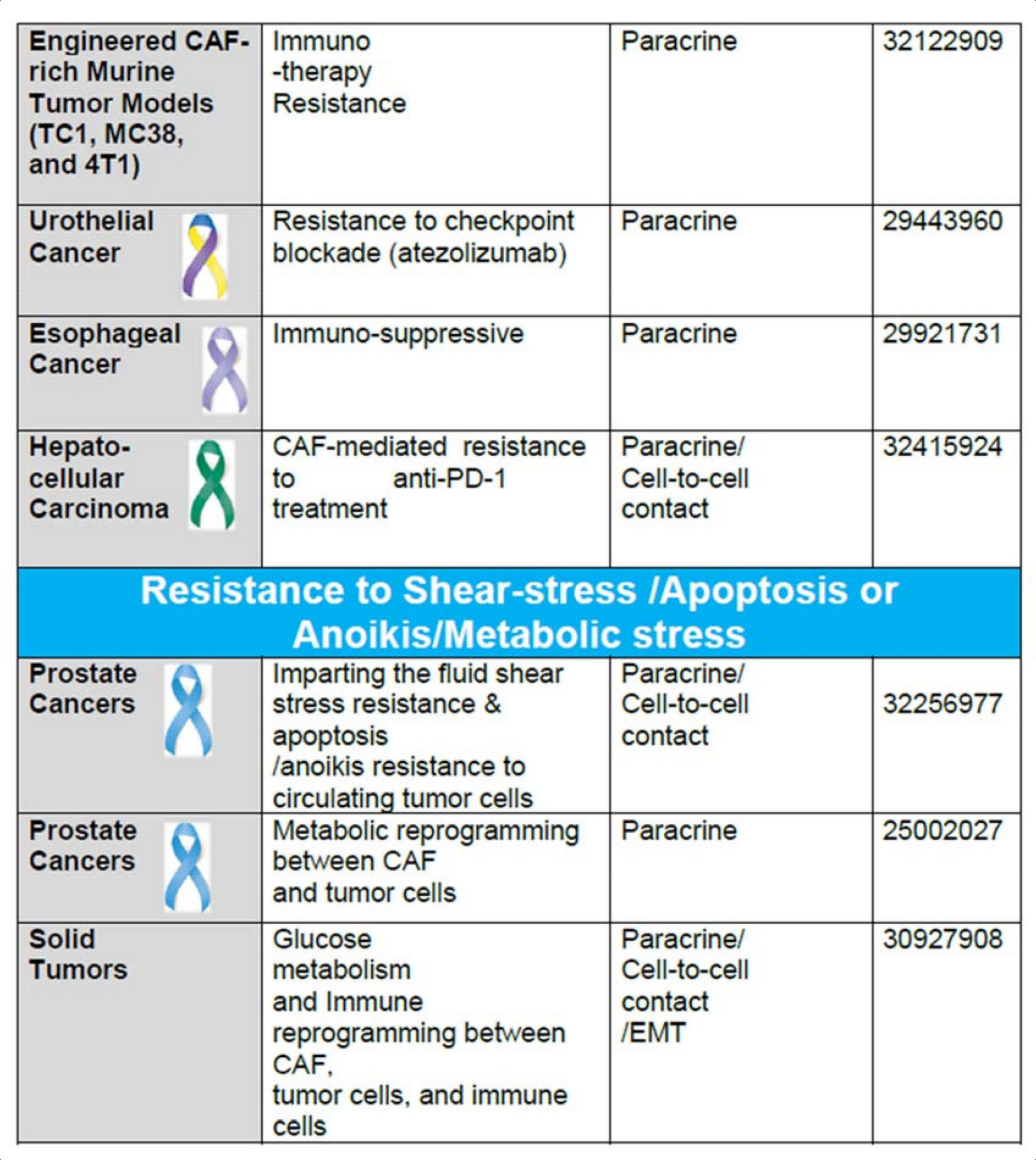
CAF and Resistance to Therapy in Solid Tumors.
